# When wax wanes: competitors for beeswax stabilize rather than jeopardize the honeyguide–human mutualism

**DOI:** 10.1098/rspb.2022.1443

**Published:** 2022-11-30

**Authors:** David J. Lloyd-Jones, James J. H. St Clair, Dominic L. Cram, Orlando Yassene, Jessica E. M. van der Wal, Claire N. Spottiswoode

**Affiliations:** ^1^ FitzPatrick Institute of African Ornithology, University of Cape Town, Cape Town 7700, South Africa; ^2^ Department of Zoology, University of Cambridge, Cambridge CB2 1TN, UK; ^3^ School of Biology, University of St Andrews, St Andrews KY16 9AJ, UK; ^4^ Mariri Environmental Centre, Concession L5 South, Niassa Special Reserve, Niassa Province, Mozambique

**Keywords:** mutualism, wax-eating, evolutionary stability, heterospecific competition, greater honeyguide, third-party species

## Abstract

Many mutualisms are exploited by third-party species, which benefit without providing anything in return. Exploitation can either destabilize or promote mutualisms, via mechanisms that are highly dependent on the ecological context. Here we study a remarkable bird–human mutualism, in which wax-eating greater honeyguides (*Indicator indicator*) guide humans (*Homo sapiens*) to wild bees' nests, in an exchange of knowledge about the location of nests for access to the wax combs inside. We test whether the depletion of wax by mammalian and avian exploiter species either threatens or stabilizes the mutualism. Using camera traps, we monitored feeding visits to wax comb made available following honey harvests. We found that greater honeyguides face competition for wax from conspecifics and nine exploiter species, five of which were not previously known to consume wax. Our results support the hypothesis that heterospecific exploiters stabilize the mutualism, because wax depletion by these competitors probably limits feeding opportunities for conspecific exploiters, favouring the early-arriving individual that guided humans to the bees’ nest. These findings highlight the importance of the ecological context of species interactions and provide further evidence for how mutualisms can persist because of, and not in spite of, exploitation by third-party species.

## 1. Introduction

Mutualisms between species are an influential element of ecological food-webs and have played an important role in the diversification and coexistence of life [1–4]. Species which form mutualistic partnerships are directly and indirectly affected by other species within their ecological community [5], and competition towards mutualists by third-party species is often highly influential in shaping the ecology and dynamics of mutualisms [6–8]. Exploitation by non-mutualists should destabilize mutualisms, or even drive one of the partners extinct through competitive exclusion [9,10]. Nonetheless, many mutualisms persist or even thrive despite exploitative behaviour, potentially due to wide variation in the costs placed on mutualists by exploiters, and the specific defensive mechanisms which have evolved against exploitation [4,11,12].

Although many mutualisms have been well studied under controlled or isolated *ex situ* conditions (e.g. between plants and mycorrhizae, [12]), to better understand the selective forces and evolutionary stability of some mutualisms, it can be informative to study mutualistic partners and their competitors in their natural ecological setting [4,6]. This is because the mechanisms by which mutualisms are resilient to exploitation from non-mutualists are highly diverse [4] and are often strongly related to both the specifics of the mutualism and to the ecological context in which they evolved (i.e. the sum of direct and indirect interactions between mutualists and their competitors, over a range of spatial and temporal scales) [4,11,13]. Furthermore, stabilizing mechanisms against exploitation are commonly affected by the level of dependence between mutualistic partners, and in particular whether the mutualism is obligate or facultative [4]. This further reinforces the value of studying mutualisms within the environment in which they evolved.

Here we studied a remarkable mutualism between humans and a wild bird species, the greater honeyguide (*Indicator indicator*), to map a guild of exploiter species, quantify the impact of these exploiters on the rewards available to mutualists, and investigate the likely consequences of these impacts for the stability of the mutualism. Greater honeyguides and humans are facultative partners in a reciprocal foraging mutualism [14,15], in which a greater honeyguide leads human honey-hunters to wild bees' nests (primarily honeybees of the subspecies *Apis mellifera scutellata*) using vocal signals supplemented by visual cues [16–19]. The birds benefit from eating the beeswax left behind after the humans harvest the honey (typically using an axe to access the nest and smoke to subdue the bees) [14,20], and the humans benefit from information about where hidden bees’ nests are located, and so from the calorific richness of honey and bee eggs, larvae and pupae (hereafter ‘bee larvae’) [16,19]. In common with other cases of human–wildlife cooperation [21], each partner both provides and receives a service (guiding to the bees' nest by the greater honeyguide, harvest of bee products by the human) and a resource (wax for the bird, honey for the human).

It has long been implicitly assumed that the human–honeyguide mutualism is stable (where it remains a frequent part of human foraging) because wax is a highly specialized food resource eaten by only a few species [22–26], such that greater honeyguides have essentially exclusive feeding access once it has been made accessible within the environment [22]. However, if other species do consume the wax, potentially attracted by conspicuous visual and acoustic cues while humans harvest a bees’ nest, and olfactory cues following the harvest, it could affect the human–honeyguide mutualism in two ways. First, competition for wax could reduce or abolish the reward to the greater honeyguide that invested time and energy in guiding the human, thus disincentivizing guiding behaviour and destabilizing the mutualism. Alternatively, increased competition may strengthen the mutualism by depriving other greater honeyguides (which did not guide the human) of opportunities to scrounge on the wax, thus increasing the marginal benefits to the guiding bird. Within nutritional mutualisms, in which one species provides its mutualistic partner with a resource or behavioural service (e.g. pollen transport between flowers) in exchange for a reward (e.g. nectar), the influence of competitors is often particularly apparent [6,7]. This is because it is relatively easy to detect the food being consumed by non-mutualists who have not incurred the costs of its production [5].

In this study, we used camera traps at natural honey-harvest sites to investigate beeswax depletion both by greater honeyguides and by heterospecific competitors. We first demonstrate the surprising level of competition that greater honeyguides face for a specialized resource, showing that a range of taxa previously unknown to eat wax, in fact regularly do so. Next, we test the predictions of two hypotheses regarding how this unexpected competition may influence the stability of the human–honeyguide mutualism. Our first hypothesis is that competition could *destabilize* the mutualism by reducing the benefits to the greater honeyguide of guiding behaviour. This hypothesis predicts that (i) competitors deplete the wax before greater honeyguides are able to feed, such that honeyguides do not obtain consistent feeding opportunities. This effect would be greatest if (ii) the most important competitors are diurnal and (iii) competitors consistently displace greater honeyguides from a wax resource. Our second hypothesis is that if honeyguides still get feeding opportunities despite heterospecific competition, then heterospecific competitors may, counterintuitively, *stabilize* the mutualism against conspecific competitors by decreasing the returns of arriving late at the resource, and therefore favour individual greater honeyguides that cooperate with humans. Specifically, this hypothesis predicts that (i) greater honeyguides should be first-arriving species after the wax has been exposed; (ii) the majority of greater honeyguide feeding events should be before other species feed (that is, that honeyguide feeding events should diminish after heterospecific competitors arrive) and (iii) late-arriving greater honeyguides miss feeding opportunities, because (iv) visits by heterospecific competitors fully deplete the wax.

## 2. Material and methods

### (a) Study site

**Figure 1.**
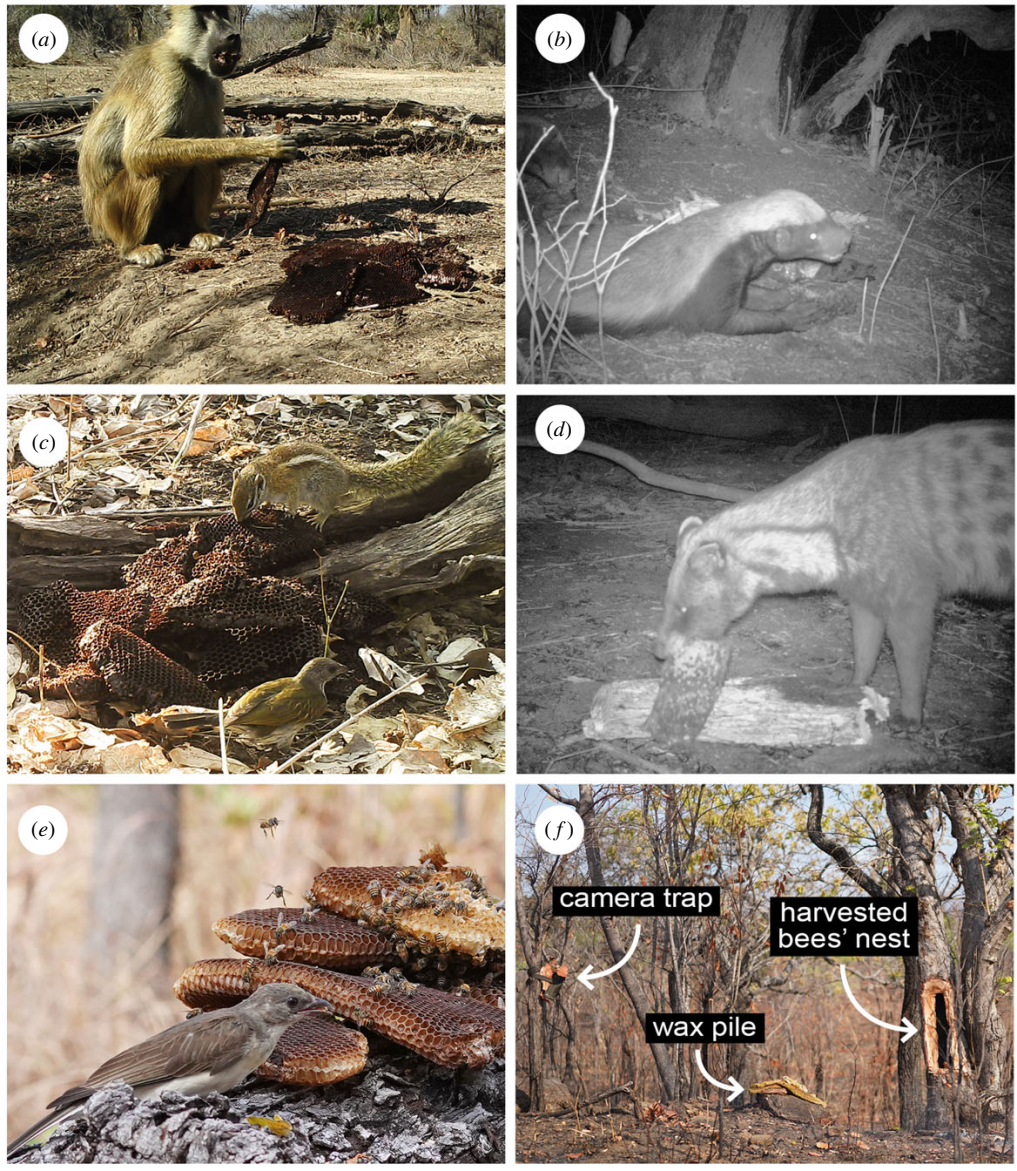
(*a*–*f*) Humans leave wax comb as a reward to greater honeyguides after a honey harvest, and this is eaten by birds and mammals. (*a*) Yellow baboon (*Papio cynocephalus*) during the day and (*b*) honey badger (*Mellivora capensis*) at night are two of the major competitors for wax which greater honeyguides (*Indicator indicator*) face. Other wax-eating animals with a lesser impact on wax availability include (*c*) striped bush squirrel (*Paraxerus flavovittis*) and scaly-throated honeyguide (*Indicator variegatus*), and (*d*) African civet (*Civettictis civetta*); (*a–d*) show camera trap images collected during the study. (*e*) An early-arriving female greater honeyguide (*Indicator indicator*) feeds on wax prior it being depleted (photograph by D.J.L.). (*f*) Photograph showing the positioning of a camera trap relative to a pile of wax and a harvested bees' nest (photograph by D.J.L.). See also electronic supplementary material, video S1.

We carried out this study in a 28 km^2^ area within the Niassa Special Reserve in northern Mozambique (electronic supplementary material, figure S1; see also [19]). Our study area is in range of Yao honey-hunters' foraging trips from Mbamba village (12°12′S, 38°01′E; *ca* 2000 inhabitants including > 20 regular honey-hunters). Yao honey-hunters traditionally reward greater honeyguides after a successful honey harvest by leaving a small pile of beeswax near the harvested bees’ nest (figure 1), and therefore the main source of wax for greater honeyguides in this landscape is that left behind or exposed by humans at the harvest site of a bees' nest [19]. Within this area, the costs and benefits of the human–honeyguide mutualism appear to approximate those under which it presumably evolved: there is little apiculture and a minimal cash economy for buying sugar instead of honey [19]. The habitat is deciduous Miombo and savannah woodland punctuated by granite inselbergs and narrow strips of riverine forest along the Lugenda River and seasonal tributaries (altitude 400–450 m). The climate is sub-humid tropical with mean minimum and maximum air temperatures ranging between 16–33°C in the dry season (May–October) and 22–32°C in the wet season (November–April). Rainfall begins in November and ends in late April or early May; during this period, precipitation averages 250–350 mm per month. Bees’ honey stores, which build up throughout the rains with the flowering of dominant species, peak in May–June, deplete as the dry season progresses and then peak again in November–December following the flowering of trees prior to the following rainy season (D.J.L, O.Y., C.N.S. 2018, 2019, personal observation) [27]. Data were collected from 24 September to 25 October 2015, 29 August to 15 October 2017, 4 November 2018, and 24 September 2021 to 7 October 2021.

### (b) Honey harvests, wax and camera trap placement

Wax-eating data were collected at 26 small piles of wax comb (approx. 0.1–1.5 kg) placed on the ground or a horizontal tree log, in a manner reflecting the honeyguide rewarding culture displayed by Yao honey-hunters (figure 1). These were located at 26 bees' nests (six nests in 2015, four in 2017 and 16 in 2021) which were found during honey-hunts conducted in a traditional manner. Eleven sites where camera traps were placed from 2015 and 2017 were excluded from the analysis either due to camera trap malfunction or because the wax was left without harvesting the bees’ nest. To initially locate the bees' nests, one or two researchers (D.J.L., C.N.S. and J.S.C.) accompanied two Yao honey-hunters (one of whom was a main assistant and is a co-author: O.Y.) on a honey-hunt as they elicited guiding behaviour from greater honeyguides using stereotypical calls [19]. Twenty-two of the 26 nests were located by guiding from a greater honeyguide, two were located opportunistically prior to being guided while walking in the same habitat and two were found in the same habitat after unsuccessful guiding by a greater honeyguide. All bees’ nest locations were previously unknown to us or the honey-hunters prior to the study, and at all 26 sites honey and wax were extracted by two honey-hunters using traditional methods (smoke and axes). The honey-hunters retained the combs containing honey and left behind a pile of wax combs without honey: these included a mixture of wax types, including smaller pieces of newly produced, empty white wax comb (most favoured by greater honeyguides), older wax comb with bee larvae in it, and dark wax comb containing old larval casings and no larvae (least favoured by greater honeyguides; D.J.L, J.J.H.S.C., D.L.C., O.Y., J.E.M.W., C.N.S. 2018, personal observation; [17]). In all cases, the wax piles were consistent with those that Yao honey-hunters naturally leave as reward following a successful harvest at a bees' nest they were guided to by a greater honeyguide.

To record animals eating visiting wax combs during the day and night, a camera trap (Acorn 6210, Ltl Acorn, Denmark, Wisconsin) was set up for 1–9 days at each site (total = 88.8 trap days; mean ± s.e. duration per site = 3.41 ± 0.39 days) at a height of 80–90 cm above ground and 1.5–2 m from the wax pile, facing slightly downwards (26.6 trap days in 2015, 15.7 trap days in 2017, 46.5 trap days in 2021). Each camera trap was set to trigger with a 5 s delay and take photos at 10 s intervals for as long as the camera was motion triggered. The cameras also recorded a video clip of 10 s or 30 s, alternating with sets of three photos for as long as the camera was motion triggered. Coordinates of the bees’ nest were marked with GPS (Garmin eTrex 30, Garmin USA) and all camera traps checked every 1–2 days.

For each animal detected, the duration of wax-feeding events was estimated from image and video clip time stamps. Feeding events were defined as contact between the mouth or bill of the animal and any part of the wax comb pile. Whenever possible we recorded which food type was eaten (wax only, larvae only, or wax and larvae together), but due to the resolution of the images we were not able to reliably score which type of wax was eaten by each animal. Short feeding events, such as when the animal disappeared prior to a second image being taken, and therefore without a reliable ‘end time’, were recorded as having a 2 s duration. At one wax site, feeding greater honeyguides disappeared to feed inside the log from which the honey and wax were harvested, and were observed emerging with small pieces of white wax. For these few observations (5 of 39 visits at this site), the period that the bird was out of view was recorded as feeding time.

### (c) Statistical analysis

All statistical analyses were carried out using R v. 4.0.3 [28]. To document the level of competition that greater honeyguides face for beeswax, we summarized the following for each wax placement site (*n* = 26): number of wax-eating visits by each species, first-arriving species and species which ate the last remaining wax (where known). Feeding times for all wax-eating species were plotted over 24 h and compared to median sunrise and sunset times (generated using the ‘suncalc’ package [29]) for our study duration).

To test prediction (i) of hypothesis one (*competitors deplete the wax before greater honeyguides feed*), we calculated the proportion of sites at which greater honeyguides fed, the frequency of feeding visits per hour and the proportion of visits which resulted in the wax pile being depleted for each species. Then, using data for the eight most frequent wax-eating species (defined as those with > 5 visits: greater honeyguide; scaly-throated honeyguide, *Indicator variegatus*; lesser honeyguide, *Indicator minor*; striped bush squirrel, *Paraxerus flavovittis*; African civet, *Civettictis civetta*; honey badger, *Mellivora capensis*; yellow baboon, *Papio cynocephalus*; Meller's mongoose, *Rhynchogale melleri*), we fitted a univariate Cox's proportional hazards survival model for arrival at wax (the event) for greater honeyguides and the other seven competitor species (pooled) using the ‘survival’ package [30]. The response term was time since wax placement, and a binary variable denoting whether the final visit time was unknown (right-censored events). The proportional hazards assumptions of this Cox regression model were met (checked by visual inspection of proportional hazard plots and tested using the cox.zph function in the ‘survival’ package; all *p* > 0.05). The results are presented as hazard ratios (HR) with corresponding 95% confidence intervals (CI).

To test prediction (ii) of hypothesis one (*most important competitors are diurnal*), we tallied the number of visits of species which consumed wax, and the time of day at which depletion (time when final piece of wax is consumed) occurred. To test prediction (iii) of hypothesis one (*diurnal competitors consistently displace greater honeyguides from a wax resource*), we first calculated the proportion of visits by diurnal competitors at wax (scaly-throated honeyguides, lesser honeyguides, striped bush squirrels, yellow baboon and slender mongoose, *Herpestes sanguineus*) which were simultaneous with greater honeyguides, then calculated the proportion of greater honeyguide feeding visits which were cut short by either of these five competitor species, and the number of greater honeyguide visits which were immediately before or after competitor species (within 10 s).

To test prediction (i) of hypothesis two (*greater honeyguides are the first-arriving species after the wax has been exposed*), we calculated the proportion of sites where greater honeyguides arrived first, along with the previous Cox's proportional hazards survival model of arrival at wax by greater honeyguides and competitor species. To test prediction (ii) of hypothesis two (*the majority of greater honeyguide feeding events occur before major wax competitors*), we first observed that the larger bodied mammals (greater than 1 kg) appearing in the images were honey badger, yellow baboon, Meller's mongoose and African civet. Preliminary observations suggested that although civets frequently visited wax sites, they were messy and often left substantial wax available, only depleting all available wax at 2 of 26 sites. For this analysis, we therefore defined the major wax competitors as yellow baboon, honey badger and Meller's mongoose, and counted the number of greater honeyguide visits to wax that occurred before or after the visits of these species, and also the number of greater honeyguide visits spent looking for pieces of wax after the wax was depleted. These counts were fitted as the response term in a generalized linear mixed effects model (GzLM) with a Poisson distribution. The number of camera trap days for each interval was included as an offset to account for variation in sampling effort, and wax placement site was included as a random term in addition to an observation level random to account for overdispersion. Similarly, we compared the feeding durations of greater honeyguides before and after the arrival of major wax competitors using a GzLM with a Gamma distribution (selected due to the data having positively skewed errors) with time spent feeding as the response term and visit interval (before major wax competitors, after major wax competitors, after wax depletion) as the predictor, and wax placement site as the random term. For both models, we report chi-squared statistics of an analysis of variance between the model of interest and the null model. Assumptions of normality for both GzLMs were assessed by visual inspection of the distribution of residuals. Effect sizes (estimated marginal means) were calculated using the ‘emmeans’ package [31]. Feeding rates for greater honeyguides were calculated by dividing the total number of feeding visits by the total number of daylight hours the wax was available for (daylength was calculated using the ‘suncal’ package [29]), over all sites. This was repeated for visits before and after major wax competitors.

We tested prediction (iii) of hypothesis two (*late-arriving greater honeyguides miss feeding opportunities*) by comparing the duration of greater honeyguide feeding visits to wax before and after major wax competitors arrived using the same GzLMs as for prediction (ii) of hypothesis two. We tested prediction (iv) of hypothesis two (*visits by heterospecific competitors fully deplete the wax)* using a Cox's proportional hazards model of wax survival with the arrival of major wax competitors (yellow baboon, Meller's mongoose and honey badger) as a time-dependant covariate. The response terms were the time until depletion after wax placement and arrival times of major wax competitors, and we included right-censored data as a binary variable (sites where the time of final wax depletion was unknown). The proportional hazards assumptions of this Cox regression model were checked as above and results presented as HR with corresponding 95% CI. Data and code used for analyses are available from the Dryad Digital Repository [32].

## 3. Results

### (a) Greater honeyguides experience surprising competition for wax from heterospecifics

**Figure 2.**
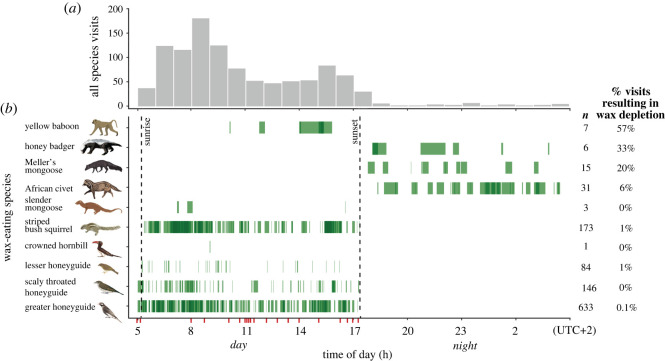
(*a,b*) Ten species were recorded eating wax yet differed in the time of day they fed and in feeding duration, and we observed a clear day–night difference in feeding visits. (*a*) Frequency of feeding visits by hour of day—each vertical bar corresponds to the total number of visits recorded within that hour; this is grouped for all days that wax was available at 26 wax sites and includes revisits by a single individual. (*b*) Green horizontal bars represent the time of day and feeding duration of visits detected at piles of wax comb and are plotted with 50% transparency such that events occurring at the same time of day appear darker. Dashed vertical lines represent the median sunrise and sunset time during the data collection periods. Red tick marks along the *x*-axis represent the wax placement times (i.e. when the wax was first available to be eaten). Total number of observed visits for each species are listed on the right (*n*). Percentage of visits for each species’ which resulted in total wax depletion is listed to far right. (Online version in colour.)

We found that 10 vertebrate species consumed beeswax (four birds, including greater honeyguides, and six mammals; figure 2). All but one species was recorded on our camera traps, yielding 1098 unique wax-eating visits. A crowned hornbill (*Tockus alboterminatus*) was observed eating wax comb (beeswax and larvae together) at a separate honey harvest in November 2018 where no camera trap was placed.

Wax-eating species differed markedly in the time of day when they fed (figure 2*a*). Four bird species (greater honeyguide, lesser honeyguide, scaly-throated honeyguide and crowned hornbill) and three mammal species (striped bush squirrel, slender mongoose and yellow baboon) were observed feeding only during daylight hours, from a median sunrise of 05 : 15 to median sunset of 17 : 25. Three larger bodied mammal species (honey badgers, African civets and Meller's mongoose) were only observed feeding on wax at night (figure 2*b*). For five species (striped bush squirrel, slender mongoose, Meller's mongoose, yellow baboon and crowned hornbill), these are the first records, to our knowledge, of wax-eating behaviour [33]. Overall, a mean ± s.e. of 4.14 ± 0.32 (median = 4, range = 1–7) wax-eating species visited each wax site (see also electronic supplementary material, figure S3). Three major wax-depleting species (yellow baboon, honey badger and Meller's mongoose) were responsible for 11 out of 20 (55%) observed wax depletions (where the final animal to finish the wax was known). When these ‘major wax competitors' fed at a wax site, the probability of all the wax being entirely depleted on that specific visit was 32%. Three mammalian wax-eating competitors (honey badger, African civet and Meller's mongoose) only fed on wax nocturnally, despite wax always becoming available diurnally (figure 2*b*). Honey badgers arrived and fed at 4/26 sites (15%), Meller's mongoose fed at 7/26 wax sites (27%) and civets arrived and fed at 12/26 wax sites (46%). These three species finished all available wax on 33%, 20% and 6% of visits, respectively. The overall proportion of wax placements depleted by sunrise on day two was 12%, and by sunrise on day three was 46%.

During the day, yellow baboons and striped bush squirrels were the primary competitors for wax, but in different ways. Yellow baboons only arrived at 7/26 wax sites (27%) yet ate all available wax on 4/7 occasions (57%) and fed for considerable periods of time (mean ± s.e. of 1375 ± 128 s) while taking the time to dextrously pick up even minute pieces of wax (electronic supplementary material, video S1), thus depriving later greater honeyguides of feeding opportunities. By contrast, striped bush squirrels made numerous visits to known wax sites (figure 2*b*, mean visits per site ± s.e. of 14.4 ± 2.9) and fed for considerable periods of time (mean ± s.e. of 293 ± 42 s) per visit. Striped bush squirrels totally depleted 2 of 20 wax sites where the final animal to feed was known (10%), yet unlike all the larger bodied competitors, did so without physically excluding greater honeyguides from access to wax.

### (b) Does competition by heterospecifics destabilize the mutualism?

**Figure 3.**
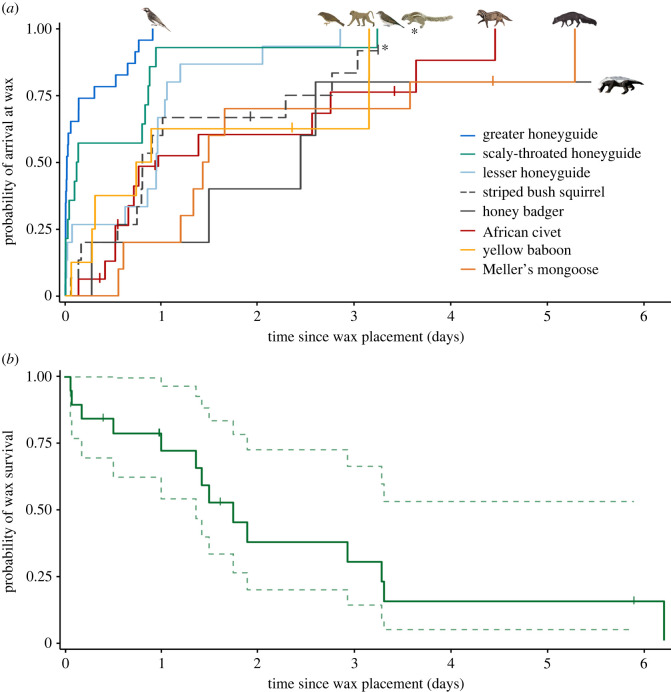
(*a,b*) Beeswax rapidly disappears when left at a honey-harvest site, due to the arrival of wax-eating mammals, yet greater honeyguides consistently arrive at wax ahead of competitor species. (*a*) Lines show the probability of arrival and wax-eating by one of the seven most common wax-eating species. These inverse Kaplan–Meier survival curves approximate the cumulative probability that a species will arrive at wax by a given time, given that they arrive at all. Steeper slopes indicate that a species, in general, finds the wax resource more quickly after it becomes exposed. Crosses mark censorship events, which are visits after the wax was depleted. (*b*) Solid green line indicates the Kaplan–Meier survival function of beeswax comb in its natural setting. Dashed pale green lines represent model predictions with 95% CIs. Crosses indicate censorship events which are sites where the times of final wax depletion were unknown. (Online version in colour.)

The first prediction of hypothesis one is that *competitors deplete the wax before greater honeyguides are able to feed*. Instead, we found that greater honeyguides were significantly more likely to arrive at wax earlier than their seven main wax-eating competitors (figure 3*a*; Cox model: HR = 4.85 [95% CI = 3.30, 7.12], *Z* = 8.04, *p* < 0.001). We observed that greater honeyguides were the first species to feed at 18 of 26 sites (69%) and successfully fed on wax at 23 of 26 wax sites (88%). Even when bees were located without the help of a greater honeyguide, this species was still the first to feed on the wax at three of four sites (75%).

The second prediction of hypothesis one is that the *most important competitors are diurnal*. Of the 20 sites at which we observed an animal depleting the wax, diurnal species depleted 65% of sites, while nocturnal species depleted 35% of sites. The key diurnal species were yellow baboon (depleting 20% of sites) and bush squirrel (depleting 10%), while the nocturnal species were Meller's mongoose (depleting 15% of sites) and honey badger (depleting 10% of sites). Importantly, when diurnal species depleted the wax, they did so on the first day at only 10% of sites, and thus even if a diurnal species depleted the wax, greater honeyguides would typically still have opportunities to feed.

The third prediction of hypothesis one is that *competitors consistently displace greater honeyguides from a wax resource.* Instead, we found that the interaction of greater honeyguides with other species at wax was relatively uncommon. It occurred primarily with scaly-throated honeyguides, which both chase and are chased away from wax by greater honeyguides (D.J.L 2017, personal observation) [34]. Lesser honeyguides were three times observed being chased away from wax by greater honeyguides, whereas striped bush squirrels were observed feeding alongside greater honeyguides 17 times with minimal agonistic behaviour. Greater honeyguide feeding visits were visibly cut short only once out of 633 feeding visits (less than 1%; by a scaly-throated honeyguide), and greater honeyguides fed simultaneously to squirrels (*n* = 17), lesser honeyguides (*n* = 11) and scaly-throated honeyguides (*n* = 22) on 50 out of 633 feeding visits (7.8%). Greater honeyguides fed immediately before or after one of these three species on 24 out of 633 visits (3.7%), indicating that while competitive displacement from wax can occur, exclusion is not widespread, and its effects are limited.

Honey badgers, civet, yellow baboon and slender mongoose all are considerably larger than honeyguides and are opportunistic bird predators [35], so would be expected to displace greater honeyguides at wax. Instead, we found that honey badgers and civets fed on wax entirely nocturnally and thus were not observed interacting with greater honeyguides (figure 2). Diurnal slender mongooses tended to arrive later to wax sites than greater honeyguides (figure 3*a*) and were less likely to arrive at wax overall: they appeared on the camera traps at 5 of 26 (19%) of wax sites but only arrived before wax depletion at 2 of 26 (7.6%) sites. Therefore, these four possible predators appear to have a minimal influence on greater honeyguides' direct access to available wax, but rather deplete the wax resource at times when greater honeyguides never or rarely visit.

### (c) Could competition by heterospecifics stabilize the mutualism against conspecific exploiters?

If greater honeyguides obtain feeding opportunities despite heterospecific competition, then heterospecific competitors may, counterintuitively, stabilize the mutualism against late-arriving conspecific exploiters (i.e. greater honeyguides that have not participated in the mutualism by guiding the human) by depriving them of wax. Hypothesis two makes four predictions, which we test using our data. First, *greater honeyguides are the first-arriving species after the wax has been exposed*. Our data provided strong support for this prediction, because greater honeyguides were the first wax-feeding species at 69% of sites (*n* = 18), and overall, they discovered wax earlier than all other species (figure 3*a*), with 56% of wax-eating visits falling within the first 24 h after wax availability.

**Figure 4.**
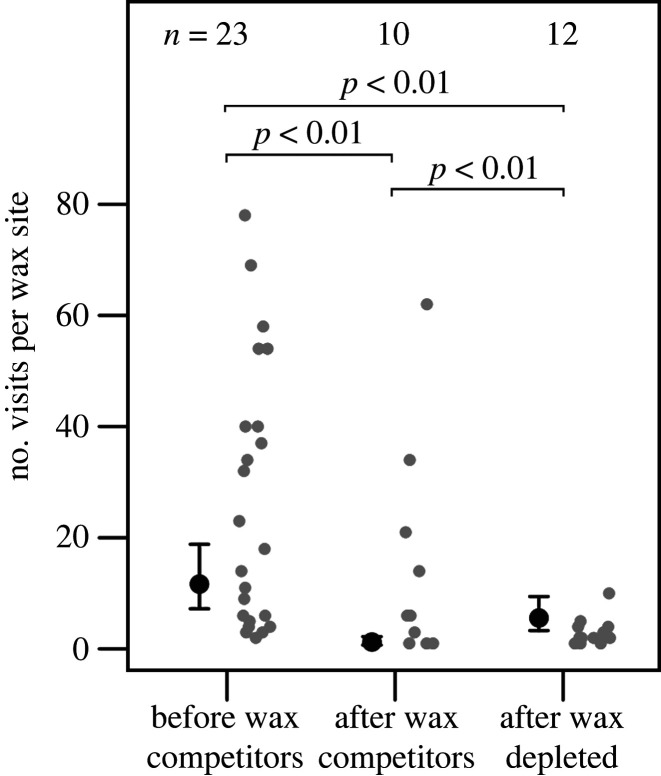
Greater honeyguides visited wax significantly more prior to the arrival of major wax competitors (yellow baboon, honey badger and Meller's mongoose) than afterwards. After all wax was depleted, greater honeyguides made more visits to search for wax than they had done when wax was still available. Black points and error bars show the predicted means and standard errors from a generalized linear mixed effects model which accounts for variability in sampling duration (i.e. how long wax was available for at each site). Grey points show the raw data. (Online version in colour.)

Second, hypothesis two predicts that *the majority of greater honeyguide feeding events occur before major wax competitors*. Our data strongly supported this prediction. We found that greater honeyguides visited wax significantly more often before the arrival of major wax competitors (yellow baboon, honey badger and Meller's mongoose) than after major wax eaters arrived, but before all wax was depleted (GzLM: *χ*^2^ = 250, d.f. = 2, *p* < 0.01, *n* = 45 counts; figure 4*a*). Number of greater honeyguides visits reduced by 89.5% after major wax competitors arrived, compared to before their arrival (0.74 ± 0.15; effect size ± standard error from GzLM; figure 4*a*). Additionally, greater honeyguide feeding rates (number of feeding visits per hour of daytime that wax was available) reduced from an average of 0.92 visits per hour to an average of 0.74 visits per hour (19.6% reduction).

Third, hypothesis two predicts that *late-arriving greater honeyguides miss feeding opportunities*. Our data provided mixed support for this prediction. Importantly, as detailed above, greater honeyguides made fewer visits to the wax after it had been visited by a major wax competitor, supporting this prediction. However, greater honeyguides that visited the wax after major wax competitors did not, as predicted, rapidly leave the site because they found no wax remaining, and instead appeared to spend longer looking for small wax pieces as well as picking apart wax comb with more larval casings and of lower wax content. Consequently, we did not find that greater honeyguides fed at or visited the wax site for significantly less time after the arrival of wax-depleting species (GzLM: *χ*^2^ = 1.65, d.f. = 2, *p* = 0.43, *n* = 783).

Finally, and critically, hypothesis two predicts that *visits by heterospecific competitors fully deplete the wax*. This prediction was strongly supported. We found that collectively, visits by the three major wax competitors (yellow baboon, Meller's mongoose and honey badger), were significantly associated in time with the complete depletion of wax (figure 3*b*: HR = 5.04 [2.11, 12.0], *Z* = 3.64, *p* < 0.01). This result is non-significant when visits by civets are included in the predictor variable, as expected since civets are not major wax competitors because they feed messily and rarely deplete the wax (Methods). The likelihood of all wax being depleted (figure 3*b*) across all sites was 19% (95% CI = 3–33%) at 24 h after it became available, 53% (95% CI = 28–70%) at 48 h and 58% (95% CI = 32–74%) at 72 h.

## 4. Discussion

Our results suggest that the observed persistence of the human–honeyguide mutualistic foraging partnership may be at least partly *due to*, and not *in spite of*, heterospecific consumption of the wax reward it produces. Prior to our results, the implicit assumption has been that greater honeyguides have essentially unrestricted feeding access to wax once it is made accessible, because few other species can digest it, such that this monopoly on wax-eating maintains the human–honeyguide mutualism. Our findings overturn these assumptions because greater honeyguides face stiff competition for wax from a surprising range of taxa, including some previously not known to eat wax. Despite this unexpected heterospecific competition for wax, our data were most consistent with the hypothesis that this competition stabilizes the mutualism against conspecific exploiters (table 1), by reducing the benefits of arriving late at the resource (and therefore favouring, at each site, the early-arriving individual greater honeyguide that cooperated with humans). This was supported by the relatively rapid removal of wax by heterospecific competitors after it first became available, and by a consequent reduction in feeding by late-arriving greater honeyguides.

**Table 1.** Competition for beeswax exposed by the human–honeyguide mutualism may play an unrecognized role in the mutualism's ecological dynamics and evolutionary stability. Our data were most consistent with the hypothesis that competition by heterospecifics stabilizes the mutualism against conspecific exploiters, by increasing the benefits to honeyguides of arriving early at the resource.

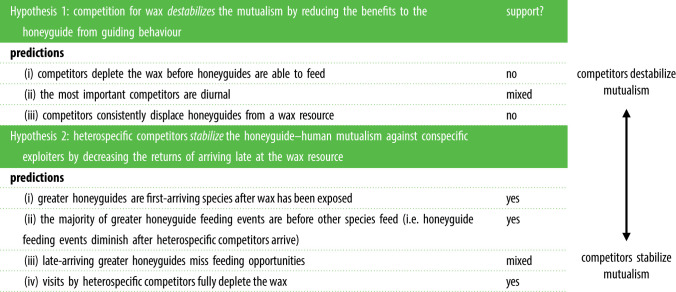

Conversely, we did not find support for the hypothesis that heterospecific competition for wax destabilizes the mutualism. Greater honeyguides were frequently able to feed on wax prior to and after other species' arrivals and even when temporarily displaced by other animals. At piles of wax not found in the daytime by yellow baboons, the two species which most consistently finished all the wax upon arrival (Meller's mongoose and honey badger) were only observed to eat wax nocturnally, therefore allowing a daytime period after a honey harvest during which greater honeyguides have opportunities to feed. Anecdotally, we also observed greater honeyguides flying away from the wax piles with large pieces of new, white wax comb, potentially to cache them (electronic supplementary material, video S1; see also [16,17]). Caching behaviour by early-arriving greater honeyguides would provide them with *ad libitum* wax for several days, while late-arriving individuals are deprived (by the early birds, and other competitors) of the new white wax which they prefer (D.J.L, O.Y, C.N.S 2015, 2017, 2018, personal observation) [20].

Our findings that greater honeyguides are typically the first species to arrive at wax, and that more than one individual arrived and fed at every site which this species visited, indicate that intraspecific dynamics are also relevant to the mutualism's stability. For example, these observations raise the question of why greater honeyguides guide humans when they can readily exploit the guiding efforts of other greater honeyguides. Answering this requires further investigation into the benefits of guiding and wax-eating at the individual level. Additionally, the interactions between greater honeyguides and scaly-throated honeyguides need further investigation; these two species are similar in size and mass (greater honeyguides in Niassa: mean mass 47.7 g, range 59.5–35.5 g, *n* = 124; scaly-throated honeyguides in Niassa: mean mass 48.1 g, range 54.5–40.5 g, *n* = 26), and do not appear capable of entirely excluding each other from wax, even when four scaly-throated honeyguides were present at the same time as one greater honeyguide (D.J.L 2017, personal observation). The probability of scaly-throated honeyguides finding wax within the first few hours after it became available is initially very similar to that of greater honeyguides (figure 3*a*), but rapidly reduces as time passes. This suggests that competitors may eavesdrop on the guiding signals from greater honeyguides to humans, as well as on the cues of a honey harvest including chopping sounds, smoke and increased bee activity.

Our results indicate that the wax liberated by the human–honeyguide mutualism has a larger ecological impact than expected. Specifically, we provide evidence this wax provides a nutritional resource for a formerly unrecognized guild of nine species, and that wax-eating by these species was remarkably common: wax at honey-harvest sites fed on average 2.7 species other than greater honeyguides. Five of the six mammalian competitor species (all but yellow baboons) readily ate empty wax combs comprised newly deposited wax. This suggests that these species may have a means of gaining energy from wax digestion, and that the competition for wax towards greater honeyguides is not simply an incidental by-product of consumption of honey or bee larvae. Alternatively, white wax may indeed be indigestible for mammalian competitors, but be consumed because it smells like comb containing honey or larvae.

These findings have several ecological and conservation implications. First, our data add to the natural histories of the nine competitor species, in six of which wax-eating has not previously been documented. Our findings suggest that the physiological ability to digest wax may be much more widespread in terrestrial species than previously thought [25]. Second, our results reveal the influence of third-party species on a functioning mutualism involving our own species and highlight how mutualisms can exert substantial and potentially cryptic ecological effects. While the effects of the human–honeyguide mutualism on local honeybee, tree and wildfire ecology remain to be quantified, our findings uncover a guild of species that regularly benefits from wax released when humans and greater honeyguides cooperate. Third, our results may have conservation implications for the human–honeyguide mutualism because protecting it may require the conservation of major wax competitor species that help stabilize it. Reciprocally, the decline of the human–honeyguide mutualism would jeopardize not only the material and non-material benefits for the two species involved [35], but may also have negative consequences for at least nine other wax-eating species.

Finally, these findings may help to explain why mutualism between greater honeyguides and humans persists across a diversity of human cultural traditions, regardless of whether or not the cooperating human culture actively rewards the guiding greater honeyguide. While the Yao honey-hunters at our study site in Mozambique consistently leave a pile of wax as a reward for the bird, other cultures (e.g. Boran [17] and Awer [36] people in Kenya, and Hadzabe, Sonjo, Maasai and Ndorobo people in Tanzania [18,37]) vary in how and when they leave a wax reward. Some honey-hunters attempt to deprive the honeyguide, reporting that hunger motivates the bird to immediately guide them to another bees' nest, and that the bird should only be rewarded—if at all—once they have harvested the day's final bees’ nest [17,37]. In doing so, honey-hunting cultures which do not always actively leave a reward may favour early-arriving birds that can clean up whatever small scraps of wax remain, producing a similar effect to major mammalian wax competitors in this study. Thus, rewarding traditions which limit wax availability may stabilize the mutualism by a different mechanism to the one that hunter-hunters envisage.

It is well understood that most mutualisms influence and are influenced by non-mutualist taxa [9,38,39]. Exploiters or cheating species are common [10] and can remain closely associated with mutualisms over long spans of evolutionary time [40,41]. Given that the ecological guild of heterospecific competitors at our study site in the Niassa Special Reserve is probably not too dissimilar to those under which the honeyguide–human mutualism first evolved [19], it is plausible that similar competition has existed over much or all of its likely ancient evolutionary history [21], and so influenced its long-term maintenance. Our finding align with recent work showing that in the cleaner–client interaction (a service-resource mutualism), the presence of third-party species directly influences the consistency (and thus stability) of the mutualism. Cleaning behaviour of brain coral by sharknose gobies *Elacatinus evelynae* was consistently more frequent when the presence of third-party species and mutualistic partner abundance locally increased [42]. Similarly, a third-party scale insect species was found to strengthen an ant–plant (resource–protection) mutualism which in turn stabilizes an entire African savannah ecosystem [43]. In the same ant–plant mutualism, the removal of an influential third-party mammal species caused mutualism breakdown [44]. These studies, together with our work and recent theoretical advances [45] collectively demonstrate the importance of third-party species to the stability of mutualisms via a range of mechanisms, including the creation of partner choice options (gobies and corals), elevation of marginal benefits to the mutualistic individuals (scale insects and ant–plant mutualism; honeyguides and humans), and increasing resilience to perturbations [45].

Overall, our results show that an unexpectedly large number of species feed on the wax resulting from the human–honeyguide mutualism, and that instead of threatening the mutualism, this ecological community probably helps to stabilize it. Thus, the human–honeyguide mutualism both supports and is maintained by a community of wax-eating species. These findings provide further evidence for how mutualistic dynamics are often context-specific and yet remain stable despite heterospecific exploitation.

## Data Availability

Data and code used in our analyses are available from the Dryad Digital Repository: https://doi.org/10.5061/dryad.d7wm37q4m [32]. Electronic supplementary material is available at Figshare [46].
